# Angiography‐Based Index of Microcirculatory Resistance in Assessing the MVO and Infarct Size in STEMI Patients

**DOI:** 10.1002/clc.70464

**Published:** 2026-09-02

**Authors:** Tian Wu, Xiaojiao Zhang, Chen Li, Yumeng Hu, Mengying Dong, Jiaqi Chai, Inam Ullah, Han Xu, Pengsheng Chen, Huiping Xu, Xiaoru Cheng, Xiaoxuan Gong, Wei Li, Jianping Xiang, Ruina Hao, Chunjian Li

**Affiliations:** ^1^ Department of Cardiology The First Affiliated Hospital with Nanjing Medical University Nanjing China; ^2^ ArteryFlow Research and Development Center for Intelligent Diagnosis and Treatment of Cardiovascular and Cerebrovascular Diseases ArteryFlow Technology Co., Ltd Hangzhou China; ^3^ Department of Cardiology Fuping County Hospital Fuping China

**Keywords:** coronary microvascular dysfunction (CMD), index of microcirculatory resistance (IMR), recombinant staphylokinase (r‐SAK), ST‐segment elevation myocardial infarction (STEMI)

## Abstract

**Objectives:**

To evaluate angiography‐based index of microcirculatory resistance (angio‐IMR) in assessing microvascular obstruction (MVO) and infarct size (IS) in ST‐segment elevation myocardial infarction (STEMI).

**Background:**

The effect of thrombolysis on post‐percutaneous coronary intervention (PCI) angio‐IMR, and its associations with MVO and IS remains unclear.

**Methods:**

One hundred twenty‐three STEMI patients randomized to receive 5 mg intravenous bolus of recombinant staphylokinase (r‐SAK) or normal saline (NS) before PCI were recruited. Angio‐IMR was computed in infarct‐related arteries. MVO and IS were detected by cardiac magnetic resonance imaging.

**Results:**

Compared with NS group, r‐SAK group exhibited numerically lower post‐PCI angio‐IMR (39.12 U vs. 42.57 U; *p* = 0.567), MVO (54.0% vs. 70.9%; *p* = 0.059), MVO extent (0.70% vs. 1.90%; *p* = 0.101) and IS (21.30% vs. 24.50%; *p* = 0.079). Post‐PCI angio‐IMR was positively correlated with MVO extent (*ρ* = 0.347; *p* < 0.001) and IS (*ρ* = 0.324; *p* < 0.001). Receiver operating characteristic analyses showed moderate diagnostic performance of angio‐IMR for MVO (area under the curve [AUC] = 0.750; *p* < 0.001), MVO > 2.6% (AUC = 0.735; *p* < 0.001) and IS > 25% (AUC = 0.712; *p* < 0.001). The exploratory optimal cut‐off values for these endpoints were approximately 40 U.

**Conclusions:**

In STEMI patients, a single bolus of r‐SAK before PCI was associated with numeric reductions in post‐PCI angio‐IMR, MVO, MVO extent and IS. Additionally, angio‐IMR exhibited a significantly positive correlation with both MVO extent and IS, demonstrating the diagnostic value of this wire‐free method for assessing microvascular injury.

## Graphical abstract

Angio‐IMR in Assessing CMD in STEMI Patients. A study of 123 OPTIMA‐5 STEMI patients found that a single bolus of half‐dose r‐SAK before primary PCI may help to improve post‐PCI angio‐IMR and reduce MVO and IS. Post‐PCI angio‐IMR also exhibited moderate diagnostic accuracy for MVO, MVO > 2.6%, and IS > 25%. Angio‐IMR = angiography‐based index of microcirculatory resistance; AUC = area under the curve; CMD = coronary microvascular dysfunction; IS = infarct size; MVO = microvascular obstruction; NS = normal saline; PCI = percutaneous coronary intervention; ROC = receiver operating characteristic; r‐SAK = recombinant staphylokinase; STEMI = ST‐elevation myocardial infarction.

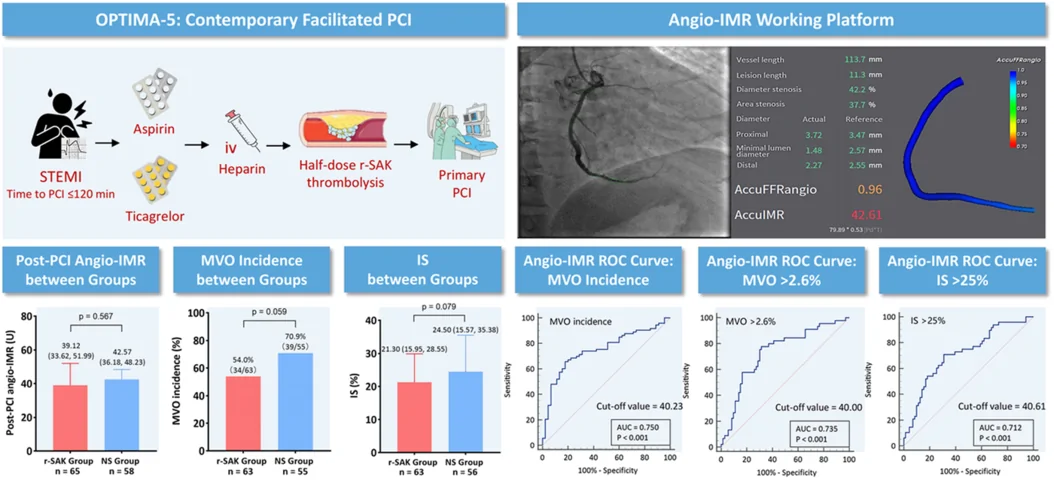

**Abbreviations**
Angio‐IMR: angiography‐based index of microcirculatory resistance
AUC: area under the curve
BMI: body mass index
CI: confidence interval
CMD: coronary microvascular dysfunction
CMR: cardiac magnetic resonance
DICOM: digital imaging and communications in medicine
ERC: Event Review Committee
IMR: index of microcirculatory resistance
IQR: interquartile range
IRA: infarct related artery
IS: infarct size
LV: left ventricular
MACE: major adverse cardiovascular events
MVO: microvascular obstruction
NPV: negative predictive value
NS: normal saline
OR: odds ratio
PCI: percutaneous coronary intervention
PPV: positive predictive value
ROC: receiver operating characteristic
r‐SAK: recombinant staphylokinase
SD: standard deviation
STEMI: ST‐elevation myocardial infarction
TIMI: thrombolysis in myocardial infarction
TVR: target vessel revascularization

## 1 Introduction

Coronary microvascular dysfunction (CMD) is a severe complication following ST‐segment elevation myocardial infarction (STEMI), with microvascular obstruction (MVO) as its central manifestation. CMD is characterized by structural and functional disturbances that impair coronary microcirculation blood flow and increase infarct size (IS) [1, 2]. Though percutaneous coronary intervention (PCI) has made significant advancements and achieved epicardial recanalization success rates of over 95%, approximately 50% of STEMI patients still develop MVO, which is significantly associated with an increased risk of major adverse cardiovascular events (MACE) [3, 4]. De et al. and Symons et al. demonstrated that MVO > 2.6% and IS > 25% were strong independent predictors of MACE in STEMI patients undergoing PCI [5, 6]. Therefore, accurate assessment of CMD is essential for early clinical intervention and improving patient prognosis.

CMD can be quantitatively evaluated through the index of microcirculatory resistance (IMR), which is calculated as the product of distal coronary pressure during maximal hyperemia and the hyperemic mean transit time [7]. In STEMI, a post‐PCI IMR > 40 U indicates severe microvascular impairment and worse clinical outcomes [8]. However, the use of invasive IMR has several limitations, such as extended procedure time, increased surgical complications, and higher costs [9]. With the advancement of computational fluid dynamics, angiography‐based IMR (angio‐IMR) has emerged as a promising wire‐free alternative to traditional IMR for detecting CMD [10, 11]. A previous study demonstrated a strong correlation between angio‐IMR and invasive IMR, with a correlation coefficient of 0.81, a sensitivity of 0.894, a specificity of 0.924, an accuracy of 0.911, and an area under the curve (AUC) of 0.92 for diagnostic efficiency [11].

The optimal management of anTIthrombolytic and throMbolytic Agent (OPTIMA)‐5 study, a prospective, multicenter, randomized trial, demonstrated that a single bolus of half‐dose recombinant staphylokinase (r‐SAK) combined with ticagrelor administered within 120 min before primary PCI for STEMI (‘contemporary facilitated PCI’) significantly improved infarct‐related artery (IRA) patency [12]. More importantly, the r‐SAK group had a lower incidence of MVO and a smaller IS based on cardiac magnetic resonance (CMR) imaging results obtained during hospitalization [12]. However, both the effect of r‐SAK thrombolysis on post‐PCI angio‐IMR and the correlation between angio‐IMR and MVO extent and IS need further investigation.

Therefore, we included patients from the OPTIMA‐5 trial to evaluate the impact of r‐SAK thrombolysis on post‐PCI angio‐IMR, MVO, MVO extent and IS, explore the correlations between angio‐IMR and MVO extent, IS, and assess the potential prognostic value of angio‐IMR in STEMI patients.

## 2 Methods

### 2.1 Study Population

This study was a retrospective analysis of patients with STEMI enrolled from the OPTIMA‐5 study, and the inclusion/exclusion criteria have been described in detail previously [12]. The study protocol, adhering to the Declaration of Helsinki and Good Clinical Practice Guidelines, was approved by the Research Ethics Board of the First Affiliated Hospital with Nanjing Medical University (approval number 2021‐SR‐309). Written informed consent was obtained from all patients prior to their inclusion in the study.

### 2.2 Angio­IMR Measurement

Angio­IMR was computed using dedicated software (AccuIMR version 1.0, ArteryFlow Technology, Hangzhou, China). All analyses were performed in a blinded manner by two trained technicians at an independent core laboratory (Guangji Core Laboratory, the Second Affiliated Hospital, Zhejiang University School of Medicine, Hangzhou, China). The technicians were blinded to all clinical data, CMR results, study group assignments and clinical outcomes. The detailed methodology was described previously [11, 13]. In brief, two angiographic views of the IRAs with at least 25° separation with minimal vessel overlap or foreshortening were selected for analyses. The lumen contour of the vessel of interest was automatically delineated and the three‐dimensional reconstruction of the vessel was generated from the delineated images. Blood flow velocity was estimated through frame count analysis, and the pressure drop across the vessel was computed based on computational fluid dynamics. Angio­IMR was defined as:

$$\mathrm{Angio}­\mathrm{IMR}=\mathrm{Pd}\times \mathrm{Tmn}=\mathrm{Pd}\times \frac{L}{V}$$

Where, *Pd* is the distal coronary pressure, *Tmn* is the mean transit time, *L* is the length of the vessel and *V* is the blood flow velocity.

### 2.3 CMR

The assessment of MVO and IS was performed by CMR scanning (3.0 T) (Philips, Ingenia, the Netherlands; Siemens, Erlangen, Germany; and GE Healthcare, Milwaukee, WI, USA) on day 5 after the enrollment. The scanning sequences included cine MR, darkblood T2 weighted imaging, T2 mapping, T1 mapping, and late gadolinium enhancement. The standard operating procedure of CMR is detailed in Supporting Information Appendix.

CMR data were recorded in digital imaging and communications in medicine (DICOM) format for subsequent analyses by an independent core laboratory blinded to all other clinical information. MVO was defined as a dark zone on early gadolinium enhancement imaging 1, 3, 5, and 7 min after contrast injection that remains present within an area of late gadolinium enhancement at 15 min [14]. IS was quantified with computer‐assisted planimetry, and the territory of infarction was delineated with the use of a signal intensity threshold of > 5 standard deviation (SD) above a remote reference region and expressed as a percentage of total left ventricular (LV) mass [15]. LV volumes and mass were normalized to body surface area, and MVO and IS were normalized to LV mass (% of LV). MVO extent was assessed in all patients, including both MVO‐positive and MVO‐negative subjects, and the MVO extent for MVO‐negative subjects was assigned as 0%.

### 2.4 Clinical Follow‐Up

Patients were followed up on days 90 ± 7, 180 ± 7, and 360 ± 14 after the enrollment through telephone calls or outpatient visits. The follow‐up endpoint was MACE, defined as a composite of all‐cause death, reinfarction, unplanned target vessel revascularization (TVR), heart failure or cardiogenic shock, and major ventricular arrhythmia. All the above‐mentioned adverse events were reviewed according to specific definitions by an independent Event Review Committee (ERC) (Supporting Information Appendix).

### 2.5 Statistical Analysis

Continuous variables were presented as mean ± SD or median (interquartile range [IQR]) and compared between groups with the Student's *t*‐test or Mann‐Whitney test as appropriate. Categorical variables were expressed as frequencies and percentages and compared using the chi‐square test or exact Fisher test. Correlations between post‐PCI angio‐IMR and MVO extent and IS were expressed with Spearman *ρ* coefficients as appropriate. The diagnostic efficacy of post‐PCI angio‐IMR was evaluated using the area under the receiver operating characteristic (ROC) curve. Univariable logistic regression was employed to calculate the unadjusted odds ratios (ORs) and 95% confidence intervals (CIs) for post‐PCI angio‐IMR to predict MVO, MVO > 2.6%, and IS > 25%. To control for potential confounders, a multivariable logistic regression model was subsequently constructed, adjusting for sex, age, use of r‐SAK, IRA, and time from symptom onset to r‐SAK or normal saline (NS) injection, thereby calculating the adjusted ORs (aORs). The Kaplan−Meier curve (tested with the log‐rank test) was used to evaluate the 1‐year MACE incidence. Patients lost to follow‐up were censored at the time of their last known alive status. A two‐sided *p* value of <0.05 was considered statistically significant. All statistical analyses were performed using MedCalc, version 20.0 (MedCalc Software Ltd., Ostend, Belgium), GraphPad Prism, version 9.0 (GraphPad Software, San Diego, CA, USA) and SPSS, version 27.0 (SPSS Inc, Chicago, IL, USA).

## 3 Results

### 3.1 Baseline Characteristics

**Figure 1:**
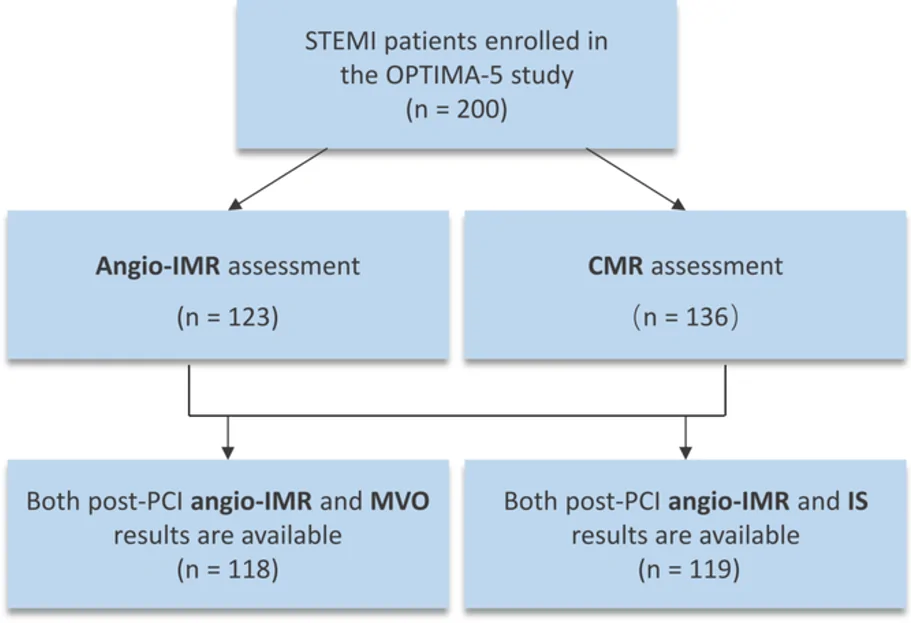
Study flow chart. Angio‐IMR = angiography‐based index of microcirculatory resistance, CMR = cardiac magnetic resonance, IS = infarct size, MVO = microvascular obstruction, PCI = percutaneous coronary intervention, STEMI = ST‐elevation myocardial infarction.

In this study, a total of 123 patients from the OPTIMA‐5 study completed angio‐IMR measurements, and 136 completed CMR examinations [12]. Among them, 118 patients had both angio‐IMR and MVO results, and 119 had both angio‐IMR and IS results (Figure 1). The baseline characteristics of these 123 patients are summarized in Table 1. Patients in the r‐SAK group had higher body mass index (BMI) values and achieved better thrombolysis in myocardial infarction (TIMI) flow grades. The distribution of baseline characteristics is consistent with that of the entire OPTIMA‐5 cohort [12]. Tables S1 and S2 present the comparison of baseline characteristics between patients with or without CMR or angio‐IMR.

**Table 1:** Baseline characteristics.

Characteristics	All (*n* = 123)	r‐SAK group (*n* = 65)	NS group (*n* = 58)	*p* value
Age, year	56 ± 10	57 ± 9	56 ± 12	0.823
Male, *n* (%)	108 (88)	55 (85)	53 (91)	0.385
BMI, kg/m^2^	24.8 (22.9, 27.1)	24.9 (23.7, 27.7)	24.2 (22.5, 26.2)	0.045
SBP, mmHg	123 ± 24	130 ± 23	125 ± 25	0.276
DBP, mmHg	80 ± 15	82 ± 14	77 ± 16	0.079
Cardiovascular risk factor, *n* (%)				
Hypertension	61 (50)	36 (55)	25 (43)	0.238
Diabetes	35 (28)	20 (31)	15 (26)	0.688
Hyperlipidemia	15 (12)	9 (14)	6 (10)	0.752
Stroke	6 (5)	3 (5)	3 (5)	1.000
Active smoking	72 (59)	39 (60)	33 (57)	0.869
Killip class, *n* (%)				0.419
Ⅰ	117 (95)	63 (97)	54 (93)	
> Ⅰ	6 (5)	2 (3)	4 (7)	
Infarct‐related artery, *n* (%)				0.473
LAD	60 (49)	29 (45)	31 (53)	
LCX	12 (10)	8 (12)	4 (7)	
RCA	51 (41)	28 (43)	23 (40)	
TIMI flow grade pre‐PCI, *n* (%)				< 0.001
0	51 (41)	14 (22)	37 (64)	
1	13 (11)	9 (14)	4 (7)	
2	18 (15)	11 (17)	7 (12)	
3	41 (33)	31 (48)	10 (17)	
TIMI flow grade post‐PCI, *n* (%)				1.000
0	0 (0)	0 (0)	0 (0)	
1	0 (0)	0 (0)	0 (0)	
2	17 (14)	9 (14)	8 (14)	
3	106 (86)	56 (86)	50 (86)	
Time from symptom to r‐SAK or NS infusion, min	230 (129, 391)	273 (137, 420)	203 (121, 357)	0.083
Thrombus aspiration, *n* (%)	17 (14)	7 (11)	10 (17)	0.437
GPI use, *n* (%)	34 (28)	16 (25)	18 (31)	0.553
LVEF, %^†^	55 (48, 60)	57 (48, 61)	54 (48, 59)	0.222
IS, % of LV^#^	22.80 (15.75, 31.70)	21.30 (15.95, 28.55)	24.50 (15.57, 35.38)	0.079
MVO, *n* (%)*	73 (62)	34 (54)	39 (71)	0.059
MVO extent, % of LV*	1.50 (0.00, 4.20)	0.70 (0.00, 3.70)	1.9 (0.00, 4.30)	0.101
Post‐PCI angio‐IMR, U	40.23 (34.92, 49.10)	39.12 (33.62, 51.99)	42.57 (36.18, 48.23)	0.567

*Note:* ^†^LVEF was measured by echocardiography using Simpson's method. ^#^Two patients in the r‐SAK group had no IS data (*n* = 63), and two patients in the NS group had no IS data (*n* = 56). *Two patients in the r‐SAK group had no MVO data (*n* = 63) and 3 patients in the NS group had no MVO data (*n* = 55). Values are mean ± SD, *n* (%), or median (25th to 75th percentile).

Abbreviations: Angio‐IMR = angiography‐based index of microcirculatory resistance, BMI = body mass index, DBP = diastolic blood pressure, GPI = platelet glycoprotein IIb/IIIa receptor inhibitor, IS = infarct size, LAD = left anterior descending artery, LCX = left circumflex artery, LV = left ventricle, LVEF = left ventricular ejection fraction, MVO = microvascular obstruction, NS = normal saline, PCI = percutaneous coronary intervention, RCA = right coronary artery, r‐SAK = recombinant staphylokinase, SBP = systolic blood pressure, SD = standard deviation, TIMI = thrombolysis in myocardial infarction.

### 3.2 Post‐PCI Angio‐IMR and MVO Between R‐SAK Group and NS Group

**Figure 2:**
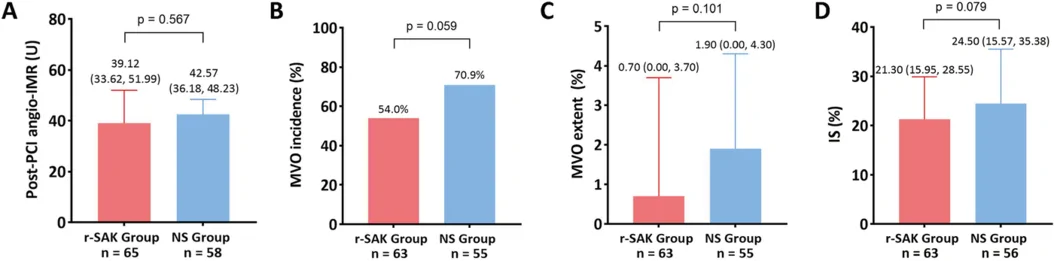
Post‐PCI Angio‐IMR, MVO, MVO Extent and IS between r‐SAK group and NS group. Bar charts showing Post‐PCI angio‐IMR (A), MVO (B), MVO extent (C), IS (D) between the r‐SAK group and the NS group. Angio‐IMR = angiography‐based index of microcirculatory resistance, IS = infarct size, MVO = microvascular obstruction, NS = normal saline, PCI = percutaneous coronary intervention, r‐SAK, recombinant staphylokinase.

The r‐SAK group demonstrated a positive trend in the following parameters though no statistically significant difference was observed: compared with the NS group, the angio‐IMR values post‐PCI were lower (39.12 U [IQR: 33.62 to 51.99 U] vs. 42.57 U [IQR: 36.18 to 48.23 U]; *p* = 0.567) (Figure 2A), the incidence of MVO was lower (54.0% vs. 70.9%; *p* = 0.059) (Figure 2B), the extent of MVO was smaller (0.70% [IQR: 0.00% to 3.70%] vs. 1.90% [IQR: 0.00% to 4.30%]; *p* = 0.101) (Figure 2C) and IS was smaller (21.30% [IQR: 15.95% to 28.55%] vs. 24.50% [IQR: 15.57% to 35.38%]; *p* = 0.079) (Figure 2D).

### 3.3 Correlations Between Post‐PCI Angio‐IMR and MVO, IS

**Figure 3:**
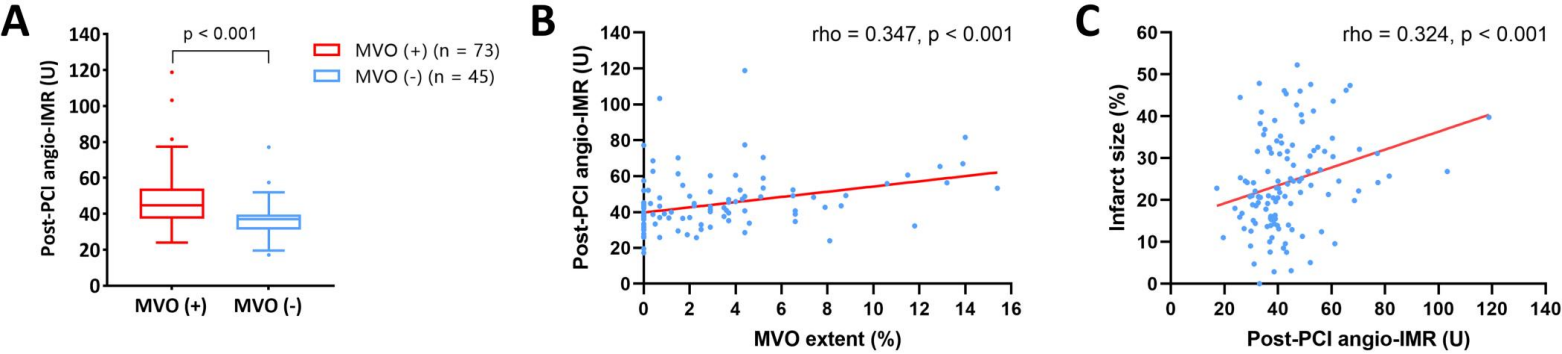
Correlations between Post‐PCI Angio‐IMR, MVO extent and IS. (A) Boxplots showing post‐PCI angio‐IMR values in patients with and without MVO. (B) Scatterplot showing correlation between post‐PCI angio‐IMR and MVO extent. (C) Scatterplot showing correlation between post‐PCI angio‐IMR and IS. Angio‐IMR = angiography‐based index of microcirculatory resistance, IS = infarct size, MVO = microvascular obstruction, PCI = percutaneous coronary intervention.

The post‐PCI angio‐IMR in the MVO‐positive group was significantly higher than that in the MVO‐negative group (44.69 U [IQR: 37.23 to 54.13 U] vs. 37.04 U [IQR: 31.25 to 39.69 U]; *p* < 0.001) (Figure 3A). Correlation analysis revealed a significantly positive correlation between post‐PCI angio‐IMR and MVO extent (*ρ* = 0.347; *p* < 0.001) (Figure 3B), as well as between post‐PCI angio‐IMR and IS (*ρ* = 0.324; *p* < 0.001) (Figure 3C).

### 3.4 Diagnostic Efficacy of Post‐PCI Angio‐IMR

**Figure 4:**
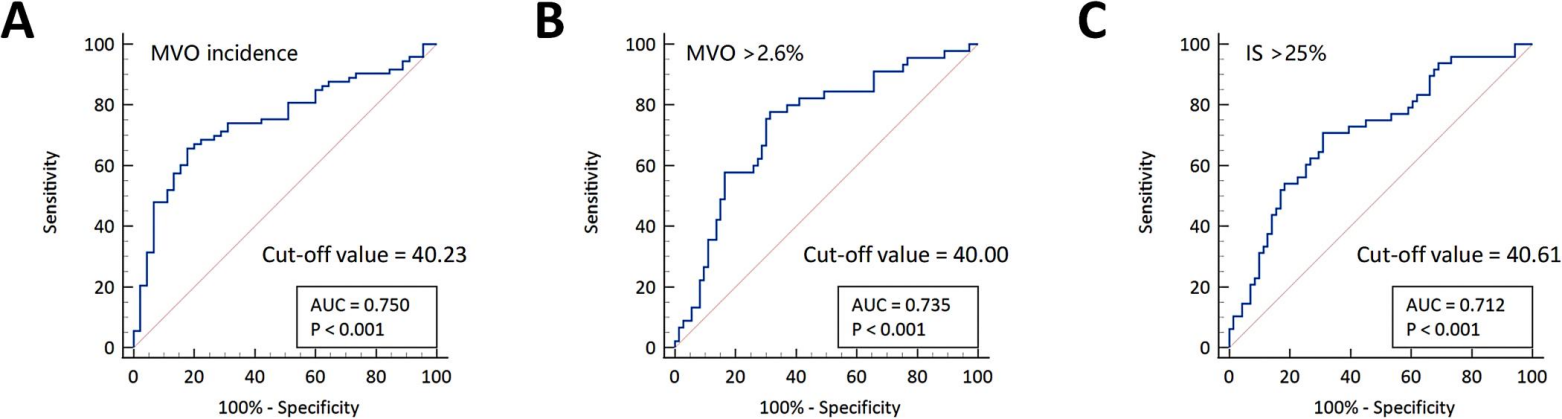
Diagnostic efficacy of post‐PCI Angio‐IMR. The ROC curve of Post‐PCI angio‐IMR to diagnose MVO (A), MVO > 2.6% (B), and IS > 25% (C). Angio‐IMR = angiography‐based index of microcirculatory resistance, AUC = area under the curve, IS = infarct size, MVO = microvascular obstruction, PCI = percutaneous coronary intervention, ROC = receiver operating characteristic.

ROC curves for post‐PCI angio‐IMR were plotted, using MVO, MVO > 2.6%, and IS > 25% as reference criteria. For predicting MVO, the optimal cut‐off was 40.23 U (AUC = 0.750, 95% CI: 0.662−0.825; *p* < 0.001), with sensitivity of 65.8%, specificity of 82.2%, Youden's index of 0.480, positive predictive value (PPV) of 85.7% and negative predictive value (NPV) of 59.7% (Figure 4A). For predicting MVO > 2.6%, the optimal cut‐off was 40.00 U (AUC = 0.735, 95% CI: 0.646−0.812; *p* < 0.001), with sensitivity of 77.8%, specificity of 68.5%, Youden's index of 0.463, PPV of 60.3% and NPV of 83.3% (Figure 4B). For predicting IS > 25%, the optimal cut‐off was 40.61 U (AUC = 0.712, 95% CI: 0.622−0.791; *p* < 0.001), with sensitivity of 70.8%, specificity of 69.0%, and Youden's index of 0.400, PPV of 60.7% and NPV of 77.8% (Figure 4C).

Based on the exploratory cut‐off of 40 U for post‐PCI angio‐IMR identified from the ROC analysis, the binary logistic regression analysis demonstrated that post‐PCI angio‐IMR > 40 U was a significant risk factor for MVO (unadjusted OR: 8.17, 95% CI: 3.39−19.66; *p* < 0.001), MVO > 2.6% (unadjusted OR: 7.61, 95% CI: 3.22−17.96; *p* < 0.001), and IS > 25% (unadjusted OR: 4.47, 95% CI: 2.03−9.85; *p* < 0.001) (Table 2).

**Table 2:** Unadjusted and adjusted binary logistic regression analysis.

	Univariate analysis		Multivariate analysis	
	Unadjusted OR (95% CI)	*p* value	Adjusted OR (95% CI)	*p* value
MVO	8.17 (3.39−19.66)	< 0.001	7.91 (3.08−20.34)	< 0.001
MVO > 2.6%	7.61 (3.22−17.96)	< 0.001	8.04 (3.15−20.53)	< 0.001
IS > 25%	4.47 (2.03−9.85)	< 0.001	4.03 (1.74−9.32)	0.001

*Note:* Adjusted models included the following covariates: sex, age, use of recombinant staphylokinase (r‐SAK), infarct‐related artery, and time from symptom onset to r‐SAK/normal saline (NS) injection.

Abbreviations: CI = confidence interval, IS = infarct size, MVO = microvascular obstruction, OR = odds ratio.

After adjusting for clinical confounding factors including sex, age, use of r‐SAK, IRA and time from symptom onset to r‐SAK or NS injection, post‐PCI angio‐IMR > 40 U still remained an independent predictor for MVO (aOR: 7.91, 95% CI: 3.08−20.34; *p* < 0.001), MVO > 2.6% (aOR: 8.04, 95% CI: 3.15−20.53; *p* < 0.001), and IS > 25% (OR: 4.03, 95% CI: 1.74−9.32; *p* = 0.001) (Table 2).

### 3.5 1‐Year MACE Incidence

**Figure 5:**
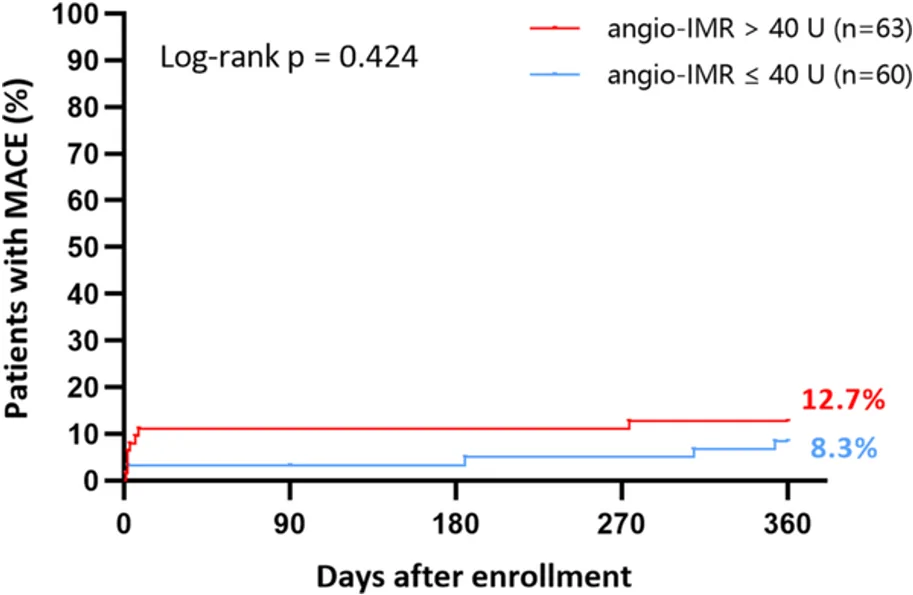
1‐Year MACE incidence. Kaplan−Meier curves showing patients with MACE during the 1‐year follow‐up. Angio‐IMR = angiography‐based index of microcirculatory resistance, MACE = major adverse cardiovascular events.

Based on the ROC analysis results from this study, an exploratory cut‐off value of 40 U for post‐PCI angio‐IMR was adopted for the survival analysis. Though not statistically significant, the Kaplan‐Meier curve demonstrated a numerically higher trend for the 1‐year MACE incidence rate in the post‐PCI angio‐IMR > 40 U group (12.7% vs. 8.3%; log‐rank *p* = 0.424) (Figure 5, Table 3).

**Table 3:** 1‐Year MACE incidence.

	Post‐PCI angio‐IMR > 40 U (*n* = 63)	Post‐PCI angio‐IMR ≤ 40 U (*n* = 60)	*p* value
MACE	8 (12.7)	5 (8.3)	0.425
All‐cause death	0 (0.0)	1 (1.7)	1.000
Reinfarction	1 (1.6)	0 (0.0)	1.000
Unplanned TVR	0 (0.0)	2 (3.3)	1.000
Heart failure or cardiogenic shock	7 (11.1)	2 (3.3)	0.055
Heart failure	6 (9.5)	1 (1.7)	0.229
Cardiogenic shock	1 (1.6)	1 (1.7)	1.000
Major ventricular arrhythmia	0 (0.0)	0 (0.0)	1.000

*Note:* Values are *n* (%).

Abbreviations: Angio‐IMR = angiography‐based index of microcirculatory resistance, MACE = major adverse cardiovascular events, PCI = percutaneous coronary intervention, TVR = target vessel revascularization.

## 4 Discussion

Angio‐IMR utilizes computational flow dynamics to derive physiological measurements from standard angiography, providing a practical method for assessing the coronary microvascular resistance, an essential parameter in evaluating CMD [13]. As a wire‐free, angiography‐derived alternative to the traditional IMR, angio‐IMR eliminates the need for pressure wires and hyperemic agents, thereby avoiding procedural delays and potential hypotensive responses [16]. This study demonstrated that the “contemporary facilitated PCI” strategy based on half‐dose r‐SAK thrombolytic therapy was associated with trends toward reduced angio‐IMR, lower incidence of MVO, smaller MVO extent and IS following PCI [12]. Notably, post‐PCI angio‐IMR showed a significantly positive correlation with both MVO extent and IS.

To our knowledge, this is the first study to investigate the impact of combining half‐dose r‐SAK thrombolysis with primary PCI on post‐PCI angio‐IMR in STEMI patients. The r‐SAK group demonstrated a positive trend in post‐PCI angio‐IMR, MVO, MVO extent and IS, although no statistically significant difference was observed. The following factors should be noted. First, the relatively small sample size may limit the statistical power to detect significant differences. Second, although the thrombotic burden in the r‐SAK group showed a favorable downward trend [12], residual thrombi might still contribute to CMD. Prior evidence indicates that even after receiving thrombolytic therapy, 18.3% of STEMI patients still harbor high‐grade residual thrombi [17].

MVO and IS, as assessed by CMR, are critical indicators of the severity of CMD and myocardial injury. However, the use of CMR is a contraindication in patients with metal implants, gadolinium allergy, claustrophobia, or severe cardiac dysfunction [18]. Our findings demonstrated significant positive correlations between post‐PCI angio‐IMR and both MVO extent and IS, suggesting that post‐PCI angio‐IMR may serve as a feasible alternative to CMR in certain circumstances. Furthermore, ROC curve analyses demonstrated that the post‐PCI angio‐IMR exhibited moderate diagnostic performance for predicting MVO, MVO > 2.6%, and IS > 25%, with the optimal cut‐off values for all three analyses converging around 40 U. This finding aligns with previous studies. Shin et al. found that the incidence of MVO was significantly higher in STEMI patients with angio‐IMR > 40 U compared to those with angio‐IMR ≤ 40 U [19]. Similarly, Choi et al. identified 40 U as the optimal threshold for diagnosing CMD in STEMI patients [20].

Beyond its value in diagnosing CMD, angio‐IMR might also demonstrate potential in predicting the long‐term clinical prognosis in STEMI patients. In our study, among patients with a post‐PCI angio‐IMR > 40 U, the incidence rates of 1‐year MACE, reinfarction and heart failure showed a numerically elevated trend.

Our results are consistent with previous studies. Kotronia et al. reported that angio‐IMR > 43 U independently predicted poor outcomes in STEMI patients, including all‐cause death, resuscitation following cardiac arrest, and heart failure (hazard ratio 2.13, 95% CI: 1.01−4.48; *p* = 0.047) [21]. Li et al. further reported that angio‐IMR > 40 U was independently associated with reduced peak oxygen uptake and an increased ventilation‐to‐carbon dioxide output slope in STEMI patients, partially explaining the underlying mechanisms of the poor clinical prognosis in this population [22]. Additionally, angio‐IMR has been proposed as an effective tool for risk stratification: a value < 40 U was associated with a lower cardiovascular risk and may help identify STEMI patients suitable for early discharge [23]. Therefore, combining the findings of this study with those from previous research may suggest that angio‐IMR has the potential to serve as a novel tool for predicting clinical outcomes in STEMI patients.

The elevated post‐PCI angio‐IMR in STEMI is more likely to be interpreted by the downstream effects of abrupt reperfusion, including capillary hyper‐pressurization, endothelial leak, edema, hemorrhage, microvascular compression, vasospasm, and distal embolization [24].

It should be noted that elevated microvascular resistance should not be presented as universally equivalent to CMD. Recent work supports an adaptive high‐resistance phenotype in stable disease when coronary flow reserve is preserved [25, 26, 27, 28]. Thus, our interpretation is particularly applicable to reperfused STEMI cases, where higher angio‐IMR values are likely indicative of acute reperfusion‐related microvascular injury.

### 4.1 Study Limitations

Despite the promising findings of this study, several unavoidable limitations should be acknowledged. First, the study is limited by a relatively small sample size, which may result in insufficient statistical power detecting the differences between groups regarding post‐PCI angio‐IMR, MVO, MVO extent, and IS. Thus, our results should be considered hypothesis‐generating, and a future validation study by a larger‐scale clinical trial is required. Second, in this study, we only focused on the angio‐IMR of IRAs in STEMI patients. Future analysis of the index of non‐IRAs in different cardiovascular scenarios is needed to further validate our results. Third, this study may not be powered enough to demonstrate the impact of angio‐IMR on patients' clinical outcomes at the 1‐year follow‐up. However, our findings could serve as a reference for future large‐scale clinical trials.

## 5 Conclusions

In STEMI patients, a single bolus of r‐SAK before PCI was associated with numeric reductions in post‐PCI angio‐IMR, MVO, MVO extent and IS. In addition, the post‐PCI angio‐IMR exhibited a significantly positive correlation with both MVO extent and IS, demonstrating the diagnostic value of this wire‐free method for assessing microvascular injury.

## Conflicts of Interest

Chunjian Li received a donation of recombinant staphylokinase and a grant from Kanion Pharmaceutical Group, Lianyungang, China. Yumeng Hu, Wei Li, and Jianping Xiang are employees of ArteryFlow Technology Co., Ltd., which provided the AccuIMR software used in this study. The other authors declare no conflicts of interest.

## Supporting information


Supporting File 1

Supporting File 2

Supporting File 3

## Data Availability

The data that support the findings of this study are available from the corresponding author upon reasonable request.
