# Supplementary material for: Angiography‐Based Index of Microcirculatory Resistance in Assessing the MVO and Infarct Size in STEMI Patients
Source: Clin Cardiol. 2026 Sep 2;49(9):e70464. doi: 10.1002/clc.70464 (PMC13535982; doi:10.1002/clc.70464)
Supplement: Supplementary file 1 [file CLC-49-e70464-s001.docx]

**Supplemental Appendix**

1. **Standard operating procedure of cardiac magnetic resonance (CMR)**

**Equipment requirements**

1. 3.0T magnetic resonance scanner and special coil for heart should be used, and the equipment shall be within the allowable service life range. Corresponding scan sequences of cardiac function, edema, scar, as well as quantitative analysis software should be available.
2. The display content should contain information including the name of the hospital, the ID of the study subject and the date of examination.

**Operators and operational requirements**

The CMR examination operator should have received formal professional training with more than 3 years of experience in CMR, and be familiar with the CMR operation and the image diagnosis.

**Scan sequences**

1. Cine MR: Balanced Steady-State Free Precession (SSFP) with a single excitation, and with retrospective electrocardiogram-triggering in 10-12 layers of short axis (SAX), covering the entire left ventricle without interval; 2ch, 3ch, 4ch long axis (LAX) collection; TR/TE: 3.5 ms/1.4 ms; reverse angle 45°; 8 mm thick; 25 frames/cardiac cycle; the spatial resolution is 2.0×1.6×8 mm^3^.
2. Dark-blood T2 weighted imaging: Short T1 Inversion Recovery (STIR) sequence, capturing 10-12 layers of SAX; TR/TE: 2 ms/75 ms; reverse angle 90°; 8 mm thick; FOV: 300×300 mm; Matrix: 160×139 mm; the spatial resolution is 1.3×1.65×8 mm^3^.
3. T2 mapping: Three layers of SAX, including basal, mid and apical, are collected by Gradient spin echo (GraSE); TR/TE: 3.3 ms/1.0 ms; reverse angle 90°; echo spacing 8.9 ms; echo number 8; the layer thickness is 8 mm and the interval is 12 mm. FOV: 340×380 mm; Matrix: 192×172 mm; the spatial resolution is 2×2×8 mm^3^.
4. T1 mapping: Modified Look-Locker Inversion recovery (MOLLI) sequence is used to collect three layers of SAX, including base, mid, and apical; TR/TE: 2.6 ms/1.2 ms; reverse angle 20°; 8 mm thick; interval of 12 mm; FOV: 340×380 mm; Matrix: 192×172 mm; the spatial resolution is 2×2×8 mm^3^.
5. Late Gadolinium Enhancement (LGE): 10 minutes after intravenous administration of 0.2 mmol/kg gadoterate magnevist (Bayer Schering Pharma AG, German); LGE is performed using 2D Phase-Sensitive Inversion Recovery (PSIR) sequence; The entire left ventricle is to be covered without interval (scan location is the same as dark-blood T2WI); Additional scanning 2ch, 3ch, and 4ch LAX images are to be obtained; TR/TE: 6.1 ms/3 ms; reverse angle 25°; 8 mm thick; FOV: 300×300 mm; Matrix: 160×139 mm; the spatial resolution is 1.9×1.6×8 mm^3^.

**Definitions of observation indexes**

1. Microvascular obstruction (MVO): MVO is defined as a dark zone on early gadolinium enhancement imaging 1, 3, 5, and 7 minutes after contrast injection that remains present within an area of late gadolinium enhancement at 15 minutes.
2. Infarct size (IS): The presence of acute infarction is to be established on the basis of abnormalities in cine wall motion by CMR, rest first-pass myocardial perfusion, and delayed-enhancement imaging in 2 imaging planes. The myocardial mass of late gadolinium (grams) is quantified with computer-assisted planimetry, and the territory of infarction is delineated with the use of a signal intensity threshold of >5 SD above a remote reference region and expressed as a percentage of total left ventricular mass.

**2. Definitions of Study Endpoints**

1. **All-cause death**

- **Cardiovascular (CV) death**(1)
  1.1 Sudden cardiac death

Death that occurs unexpectedly in a previously stable patient and includes the following deaths:

a) Witnessed and instantaneous without new or worsening symptoms;
b) Witnessed within 60 minutes of the onset of new or worsening cardiac symptoms;
c) Witnessed and attributed to an identified arrhythmia (eg, captured on electrocardiogram (ECG) recording or witnessed on a monitor by either a medic or paramedic);
d) Subjects unsuccessfully resuscitated from cardiac arrest or successfully resuscitated from cardiac arrest but who die within 24 hours without identification of a non-cardiac etiology;
e) Unwitnessed death and there is no conclusive evidence of another, non-CV, cause of death (ie, presumed CV death).
1.2 Sudden death due to acute myocardial infarction (MI)

Sudden death occurring up to 14 days after a documented acute MI (verified either by the universal definition of acute MI(2) or by autopsy findings showing recent MI or recent coronary thrombus) and where there is no conclusive evidence of another cause of death. If death occurs before biochemical confirmation of myocardial necrosis can be obtained, adjudication should be based on clinical presentation and ECG evidence.

1.3 Death due to heart failure (HF) or cardiogenic shock

Death occurring in the context of clinically worsening symptoms and/or signs of congestive HF without evidence of another cause of death.

New or worsening signs and/or symptoms of HF include any of the following:

a) New or increasing symptoms and/or signs of HF requiring the initiation of, or an increase in, treatment directed at HF or occurring in a patient already receiving maximal therapy for HF;
b) HF symptoms or signs requiring continuous intravenous therapy or oxygen administration;
c) Confinement to bed predominantly due to HF symptoms;
d) Pulmonary edema sufficient to cause tachypnea and distress not occurring in the context of an acute MI or as the consequence of an arrhythmia occurring in the absence of worsening HF;
e) Cardiogenic shock not occurring in the context of an acute MI or as the consequence of an arrhythmia occurring in the absence of worsening HF.

- Cardiogenic shock is defined as systolic pressure (SBP) <90 mmHg for more than 1 hour, not responsive to fluid resuscitation and/or heart rate correction, and felt to be secondary to cardiac dysfunction and associated with at least one of the following signs of hypoperfusion:
- Cool, clammy skin
- Oliguria (urine output <30 mL/h)
- Altered sensorium
- Cardiac index <2.2 L/min/m^2^
- Cardiogenic shock can also be defined in the presence of SBP ≥90 mmHg or for a time period <1 hour if the blood pressure measurement or the time period is influenced by the presence of positive inotropic or vasopressor agents alone and/or with mechanical support <1 hour.
  1.4 Death due to stroke, cerebrovascular events

Death occurring up to 30 days after a stroke that is either due to the stroke or caused by complication of the stroke.

1.5 Death due to other CV causes

Death must be due to a fully documented CV cause not included in the above categories (eg, dysrhythmia, pulmonary embolism, or CV intervention). Death due to a MI that occurs as a direct consequence of a CV investigation/procedure/operation will be classified as death due to other CV cause. In addition, death due to hemorrhage will be included.(3)

1.6 Death due to unknown cause(4)

These are expected to be rare and mostly when there is no supportive documentation available despite several requests.

- **Non-CV death**(1)

Non-CV death is defined as any death not covered by cardiac death or vascular death. Examples of non-CV death are: pulmonary causes, renal causes, gastrointestinal causes, infection (including sepsis), non-infectious (eg, systemic inflammatory response syndrome), malignancy (ie, new malignancy, worsening of prior malignancy), accidental/trauma, suicide, non-CV organ failure (eg, hepatic failure) or non-CV surgery.

2. **Reinfarction**
   2.1 Criteria for acute MI (types 1, 2 and 3 MI)(2)

The term acute MI should be used when there is acute myocardial injury with clinical evidence of acute myocardial ischemia and with detection of a rise and/or fall of cardiac troponin (cTn) values with at least one value above the 99th percentile upper reference limit (URL) and at least one of the following:

a) Symptoms of myocardial ischemia;
b) New ischemic ECG changes;
c) Development of pathological Q waves;
d) Imaging evidence of new loss of viable myocardium or new regional wall motion abnormality in a pattern consistent with an ischemic etiology;
e) Identification of a coronary thrombus by angiography or autopsy (not for types 2 or 3 MIs).

Post-mortem demonstration of acute atherothrombosis in the artery supplying the infarcted myocardium meets criteria for type 1 MI. Evidence of an imbalance between myocardial oxygen supply and demand unrelated to acute atherothrombosis meets criteria for type 2 MI. Cardiac death in patients with symptoms suggestive of myocardial ischemia and presumed new ischemic ECG changes before cTn values become available or abnormal meets criteria for type 3 MI.

2.2 Criteria for coronary procedure-related MI (types 4 and 5 MI)(2)

a) Percutaneous coronary intervention (PCI) related MI is termed type 4a MI.
b) Coronary artery bypass grafting (CABG) related MI is termed type 5 MI.

Coronary procedure-related MI ≤48 hours after the index procedure is arbitrarily defined by an elevation of cTn values >5 times for type 4a MI and >10 times for type 5 MI of the 99th percentile URL in patients with normal baseline values. Patients with elevated pre-procedural cTn values, in whom the pre-procedural cTn level are stable (≤20% variation) or falling, must meet the criteria for a >5 or >10-fold increase and manifest a change from the baseline value of >20%. In addition, with at least one of the following:

a) New ischemic ECG changes (this criterion is related to type 4a MI only);
b) Development of new pathological Q waves;
c) Imaging evidence of loss of viable myocardium that is presumed to be new and, in a pattern, consistent with an ischemic etiology;
d) Angiographic findings consistent with a procedural flow-limiting complication such as coronary dissection, occlusion of a major epicardial artery or graft, side-branch occlusion-thrombus, disruption of collateral flow or distal embolization.
   2.3 Within the first 24 hours after randomization (5)

Reinfarction is defined as:

a) New ischemic symptoms greater than 20 minutes,

**AND**

b) New or recurrent ST segment elevation or depression >0.1 millivolt in >2 contiguous leads.
   2.4 Between 24 hours and 7 days after randomization(5)

Reinfarction is defined as ischemic symptoms greater than 20 minutes,

**AND**

a) Re-elevation of cTn to above the upper limit of normal and increased by ≥50% over the previous value;(6)

**OR**

b) New or recurrent ST segment elevation or depression >0.1 millivolt or new significant Q-waves in >2 contiguous leads discrete from the baseline MI.
   2.5 After 7 days of randomization(5)

Reinfarction is defined as 3.1 and 3.2.

3. **Unplanned target vessel revascularization** **(TVR)**(7)

Unplanned TVR is defined as any unexpected repeat percutaneous intervention or surgical bypass of any segment of the target vessel. The target vessel is defined as the entire major coronary vessel proximal and distal to the target lesion, which includes upstream and downstream branches and the target lesion itself.

4. **Heart failure and cardiogenic shock**
   4.1 Heart failure(6, 8)

The patient exhibits documented new or worsening symptoms due to HF on presentation, including at least one of the following:

a) Dyspnea (dyspnea with exertion, dyspnea at rest, orthopnea, paroxysmal dyspnea);
b) Decreased exercise tolerance;
c) Fatigue;
d) Other symptoms of worsened end-organ perfusion such as dizziness, mental confusion or volume overload such as weight gain or lower extremity swelling.

**AND**

The patient has one of the following objective evidence of new or worsening HF, requiring treatment with diuretics:

a) Pulmonary edema/congestion on chest X-ray or computed tomography (CT) without suspicion of a non-cardiac cause;
b) Rales >1/3 up from the lung base (Killip class 2 or higher);
c) Pulmonary capillary wedge pressure (PCWP) >25 mmHg;
d) PO_2_ <80 mmHg or O_2_ sat <90% (no supplemental O_2_) in the absence of known lung disease;
e) Increased B-type natriuretic peptide (BNP)/N-terminal proBNP (NT-proBNP) concentrations consistent with decompensation of HF.
   4.2 Cardiogenic shock (defined as one of the following)(6)
a) SBP <90 mmHg for at least 30 minutes (or the need for vasopressor agents and/or mechanical circulatory support to maintain a SBP of >90 mmHg) in the presence of a heart rate of >60 beats/min in association with signs of end organ hypoperfusion (cold extremities, low urinary output <30 mL/h and/or mental confusion);
b) A cardiac index <2.2 L/min/m^2^ in the presence of a PCWP of >15 mmHg.
5. **Major ventricular arrhythmias**(9)

Ventricular arrhythmias >6 hours after randomization requiring electrical cardioversion/defibrillation.

3. **Events Review Committee**

This study adopts the method of verifying each event by a relevant professional expert, as follows:

A. The auditor agrees with the original researcher’s event report

- Indicate agreement or uncertainty on the event verification report
- The event verification database is updated by the verification assistant
- The original researcher’s judgment of the event is accepted
- Verification concludes

B. The first auditor disagrees with the original researcher’s event report

- Indicate disagreement on the event verification report
- The auditor must report a new classification or diagnosis of the event based on their assessment of the verification data
- The event verification data and new event verification report are sent to a second auditor who is blind to the previous verification results for review

C. If the second auditor agrees with the original researcher’s judgment

- Indicate agreement on the event verification report
- The original researcher’s judgment of the event is accepted, and the first auditor’s opinion is rejected
- The event verification database is updated by the verification assistant
- Verification concludes

D. If the second auditor disagrees with the original researcher but agrees with the first auditor’s determination

- Indicate disagreement on the event verification report
- The original researcher’s judgment is rejected while the first auditor’s judgment is accepted
- The event verification database is updated by the verification assistant
- Verification concludes

E. If the second auditor is uncertain about the classification

- Indicate uncertainty on the event verification report
- Both the first and second auditors will discuss the event to reach a consensus

a) If both auditors agree with the original researcher’s event report:

- Indicate agreement on the event verification report
- The original researcher’s judgment of the event is accepted
- The event verification database is updated by the verification assistant
- Verification concludes

b) If both auditors disagree with the original researcher’s event report:

- Indicate disagreement on the event verification report
- The first auditor’s judgment of the event is accepted
- The event verification database is updated by the verification assistant
- Verification concludes

c) If the first and second auditors’ opinions are inconsistent:

- Note the lack of consensus
- The event is escalated to the chair of the verification committee for a ruling (agreeing or disagreeing with the original researcher’s opinion)
- The event verification database is updated by the verification assistant
- Verification concludes

The independent ERC is composed as follows:

| **Name** | **Department** | **Medical Department** |
| --- | --- | --- |
| Jun Zhu | Cardiology | Fuwai Hospital and National Center for Cardiovascular Diseases, Chinese Academy of Medical Sciences and Peking Union Medical College |
| Xinli Li | Cardiology | The First Affiliated Hospital with Nanjing Medical University |
| Ting Wu | Neurology | The First Affiliated Hospital with Nanjing Medical University |
| Jianqing Ge | Neurology | Nanjing Brain Hospital Affiliated with Nanjing Medical University |
| Chengchun Tang | Interventional Cardiology | Zhong-Da Hospital Affiliated with South-East University |
| Jie Song | Interventional Cardiology | Gu-Lou Hospital Affiliated with Nanjing University |
| Mingfang Li | Cardiology | The First Affiliated Hospital with Nanjing Medical University |

**References**

1.	Sarafidis PA, Tsapas A: Empagliflozin, Cardiovascular Outcomes, and Mortality in Type 2 Diabetes. *N Engl J Med*. 2016;**374**(11):1092.

2.	Thygesen K, Alpert JS, Jaffe AS, Chaitman BR, Bax JJ*, et al.*: Fourth Universal Definition of Myocardial Infarction (2018). *Circulation*. 2018;**138**(20):e618-e651.

3.	Harloff A, Schlachetzki F: Rivaroxaban for Stroke Prevention after Embolic Stroke of Undetermined Source. *N Engl J Med*. 2018;**379**(10):986-987.

4.	Connolly SJ, Eikelboom JW, Bosch J, Dagenais G, Dyal L*, et al.*: Rivaroxaban with or without aspirin in patients with stable coronary artery disease: an international, randomised, double-blind, placebo-controlled trial. *Lancet*. 2018;**391**(10117):205-218.

5.	Mehta SR, Granger CB, Boden WE, Steg PG, Bassand JP*, et al.*: Early versus delayed invasive intervention in acute coronary syndromes. *N Engl J Med*. 2009;**360**(21):2165-2175.

6.	Armstrong PW, Gershlick AH, Goldstein P, Wilcox R, Danays T*, et al.*: Fibrinolysis or primary PCI in ST-segment elevation myocardial infarction. *N Engl J Med*. 2013;**368**(15):1379-1387.

7.	Cutlip DE, Windecker S, Mehran R, Boam A, Cohen DJ*, et al.*: Clinical end points in coronary stent trials: a case for standardized definitions. *Circulation*. 2007;**115**(17):2344-2351.

8.	Packer M, Anker SD, Butler J, Filippatos G, Pocock SJ*, et al.*: Cardiovascular and Renal Outcomes with Empagliflozin in Heart Failure. *N Engl J Med*. 2020;**383**(15):1413-1424.

9.	Armstrong PW, Committee WS: A comparison of pharmacologic therapy with/without timely coronary intervention vs. primary percutaneous intervention early after ST-elevation myocardial infarction: the WEST (Which Early ST-elevation myocardial infarction Therapy) study. *Eur Heart J*. 2006;**27**(13):1530-1538.
