# Supplementary material for: Angiography‐Based Index of Microcirculatory Resistance in Assessing the MVO and Infarct Size in STEMI Patients
Source: Clin Cardiol. 2026 Sep 2;49(9):e70464. doi: 10.1002/clc.70464 (PMC13535982; doi:10.1002/clc.70464)
Supplement: Supplementary file 2 [file CLC-49-e70464-s002.docx]

**Table S1 Comparison of Baseline Characteristics Between Patients with or without CMR.**

| **Characteristics** | **CMR**  **(n = 136)** | **Non-CMR**  **(n = 64)** | ***P* Value** |
| --- | --- | --- | --- |
| r-SAK group, n (%) | 75 (55) | 25 (39) | 0.034 |
| Age, y | 58 (50, 65) | 61 (51, 68) | 0.052 |
| Male, n (%) | 118 (87) | 54 (84) | 0.814 |
| BMI, kg/m^2^ | 24.80  (22.89, 27.36) | 24.73  (22.86, 27.41) | 0.637 |
| SBP, mmHg | 129 ± 25 | 124 ± 22 | 0.172 |
| DBP, mmHg | 80 ± 15 | 77 ± 17 | 0.378 |
| Cardiovascular risk factor, n (%) |  |  |  |
| Hypertension | 70 (51) | 38 (59) | 0.371 |
| Diabetes | 39 (29) | 15 (23) | 0.543 |
| Hyperlipidemia | 17 (12) | 9 (14) | 0.935 |
| Stroke | 6 (4) | 6 (9) | 0.289 |
| Active smoking | 77 (57) | 39 (61) | 0.824 |
| Killip class, n (%) |  |  | 0.022 |
| Ⅰ | 130 (96) | 55 (86) |  |
| > Ⅰ | 6 (4) | 9 (14) |  |
| Infarct-related artery, n (%)* |  |  | 0.625 |
| LAD | 65 (48) | 26 (41) |  |
| LCX | 16 (12) | 10 (16) |  |
| RCA | 54 (40) | 25 (39) |  |
| TIMI flow grade pre-PCI, n (%) |  |  | 0.050 |
| 0 | 53 (39) | 35 (55) |  |
| 1 | 13 (10) | 1 (2) |  |
| 2 | 19 (14) | 10 (16) |  |
| 3 | 51 (38) | 18 (28) |  |
| TIMI flow grade post-PCI, n (%) |  |  | < 0.001 |
| 0 | 0 (0) | 4 (6) |  |
| 1 | 0 (0) | 3 (5) |  |
| 2 | 18 (13) | 12 (19) |  |
| 3 | 118 (87) | 45 (70) |  |
| Time from symptom to r-SAK or NS infusion, min | 252 (134, 426) | 253 (188, 398) | 0.867 |
| Thrombus aspiration, n (%) | 17 (12) | 12 (19) | 0.339 |
| GPI use, n (%) | 37 (27) | 15 (23) | 0.694 |
| LVEF, %† | 55 (48, 60) | 55 (47, 61) | 0.602 |

*3 patients in the non-CMR group had no infarct-related artery data (n = 61): 1 died before coronary angiography, 1 were diagnosed as myocarditis, and 1 was diagnosed as atrial sarcoma. 1 patient in the CMR group had no infarct-related artery data (n = 135), who was diagnosed as myocarditis.

†LVEF was measured by echocardiography using Simpson’s method.

Values are mean ± SD, n (%), or median (25th to 75th percentile).

BMI = body mass index; CMR = cardiac magnetic resonance; DBP = diastolic blood pressure; GPI = platelet glycoprotein IIb/IIIa receptor inhibitor; LAD = left anterior descending artery; LCX = left circumflex artery; LVEF = left ventricular ejection fraction; NS = normal saline; PCI = percutaneous coronary intervention; RCA = right coronary artery; r-SAK = recombinant staphylokinase; SBP = systolic blood pressure; SD = standard deviation; TIMI = thrombolysis in myocardial infarction.
