# Supplementary material for: Angiography‐Based Index of Microcirculatory Resistance in Assessing the MVO and Infarct Size in STEMI Patients
Source: Clin Cardiol. 2026 Sep 2;49(9):e70464. doi: 10.1002/clc.70464 (PMC13535982; doi:10.1002/clc.70464)
Supplement: Supplementary file 3 [file CLC-49-e70464-s003.docx]

**Table S2** **Comparison of Baseline Characteristics Between Patients with or without Angio-IMR.**

| **Characteristics** | **Angio-IMR**  **(n = 123)** | **Non-angio-IMR**  **(n = 77)** | ***P* Value** |
| --- | --- | --- | --- |
| r-SAK group, n (%) | 65 (53) | 35 (45) | 0.383 |
| Age, y | 58 (50, 64) | 60 (51, 68) | 0.078 |
| Male, n (%) | 108 (88) | 64 (83) | 0.471 |
| BMI, kg/m^2^ | 24.80 (22.89, 27.11) | 24.77 (22.86, 27.43) | 0.923 |
| SBP, mmHg | 128 ± 24 | 126 ± 24 | 0.552 |
| DBP, mmHg | 79 ± 14 | 78 ± 18 | 0.485 |
| Cardiovascular risk factor, n (%) |  |  |  |
| Hypertension | 61 (50) | 47 (61) | 0.151 |
| Diabetes | 35 (28) | 19 (25) | 0.673 |
| Hyperlipidemia | 15 (12) | 11 (14) | 0.832 |
| Stroke | 6 (5) | 6 (8) | 0.542 |
| Active smoking | 72 (59) | 44 (57) | 0.962 |
| Killip class, n (%) |  |  | 0.133 |
| Ⅰ | 117 (95) | 68 (88) |  |
| > Ⅰ | 6 (5) | 9 (12) |  |
| Infarct-related artery, n (%)* |  |  | 0.168 |
| LAD | 60 (49) | 31 (42) |  |
| LCX | 12 (10) | 14 (19) |  |
| RCA | 51 (41) | 28 (38) |  |
| TIMI flow grade pre-PCI, n (%) |  |  | 0.093 |
| 0 | 51 (41) | 37 (48) |  |
| 1 | 13 (11) | 1 (1) |  |
| 2 | 18 (15) | 11 (14) |  |
| 3 | 41 (33) | 28 (36) |  |
| TIMI flow grade post-PCI, n (%) |  |  | 0.004 |
| 0 | 0 (0) | 4 (5) |  |
| 1 | 0 (0) | 3 (4) |  |
| 2 | 17 (14) | 13 (17) |  |
| 3 | 106 (86) | 57 (74) |  |
| Time from symptom to r-SAK or NS infusion, min | 230 (129, 391) | 275 (200, 461) | 0.045 |
| Thrombus aspiration, n (%) | 17 (14) | 12 (16) | 0.890 |
| GPI use, n (%) | 34 (28) | 18 (23) | 0.615 |
| LVEF, %† | 55 (48, 60) | 55 (48, 61) | 0.870 |

*4 patients in the non-angio-IMR group had no infarct-related artery data (n = 73): 1 died before coronary angiography, 2 were diagnosed as myocarditis, and 1 was diagnosed as atrial sarcoma.

†LVEF was measured by echocardiography using Simpson’s method.

Values are mean ± SD, n (%), or median (25th to 75th percentile).

Angio-IMR = angiography-based index of microcirculatory resistance; BMI = body mass index; DBP = diastolic blood pressure; GPI = platelet glycoprotein IIb/IIIa receptor inhibitor; LAD = left anterior descending artery; LCX = left circumflex artery; LVEF = left ventricular ejection fraction; NS = normal saline; PCI = percutaneous coronary intervention; RCA = right coronary artery; r-SAK = recombinant staphylokinase; SBP = systolic blood pressure; SD = standard deviation; TIMI = thrombolysis in myocardial infarction.
